# A Case of Laparoscopic Left Hemicolectomy for Transverse Colon Cancer With Severe Obesity Performed Safely by Multidisciplinary Perioperative Management

**DOI:** 10.7759/cureus.71401

**Published:** 2024-10-13

**Authors:** Yuki Matsumi, Satoru Kikuchi, Ryohei Shoji, Fuminori Teraishi, Toshiyoshi Fujiwara

**Affiliations:** 1 Department of Gastroenterological Surgery, Graduate School of Medicine, Dentistry and Pharmaceutical Sciences, Okayama University, Okayama, JPN

**Keywords:** colorectal cancer, laparoscopic colectomy, perioperative management, severe obesity, weight loss treatment

## Abstract

A minimally invasive approach using laparoscopy or robotics has become the standard procedure in surgery for colorectal cancer. However, obesity is considered to be associated with a poor prognosis in laparoscopic colorectal surgery. Perioperative management, as well as the surgical procedure, is particularly important in severely obese patients. A case of colon cancer with severe obesity that underwent laparoscopic colectomy and was managed safely by multidisciplinary perioperative management in collaboration with a bariatric and metabolic surgery (BMS) team is presented.

The patient was severely obese, with a body mass index (BMI) of 50.4 kg/m^2^. After one month of preoperative weight loss intervention by the BMS team, the patient’s weight was successfully decreased by approximately 15 kg (BMI: 46 kg/m^2^), and the patient underwent laparoscopic colectomy for transverse colon cancer in collaboration with the BMS team.

In the laparoscopic surgery, a small incision for specimen removal was made above the umbilicus to insert the first trocar safely, and five additional trocars, whose placement was determined based on the target vessels of the dissected lymph nodes in reference to preoperative computed tomography (CT), were also inserted above the umbilicus. Gastrointestinal reconstruction was performed intracorporeally by an overlap technique using an endoscopic linear stapler to perform the procedure safely with minimal invasiveness.

The patient was discharged on postoperative day eight without any postoperative complications, following early postoperative rehabilitation with intervention by the BMS team. The proportion of colorectal cancer patients with obesity is expected to increase in the future, and the establishment of multidisciplinary perioperative management and surgical techniques will be useful to improve the surgical outcomes and prognosis of colorectal cancer patients with severe obesity.

## Introduction

Minimally invasive surgery using laparoscopy or robotics has become the standard approach for colorectal cancer, offering numerous advantages such as reduced postoperative pain, shorter recovery times, and minimized scarring. However, despite these benefits, the management of obese patients undergoing such procedures presents unique challenges. According to the JCOG 0404 study, obesity, defined as a body mass index (BMI) of 25 kg/m² or more, has been associated with poorer prognoses in colorectal cancer patients, including increased rates of recurrence and complications [1,2].

The Japanese guidelines for colorectal cancer treatment emphasize that surgical decisions for obese patients must consider their higher likelihood of conversion to open surgery, extended operation times, and increased complication rates. These factors necessitate a more cautious and tailored approach to treatment. Preoperative weight loss interventions combined with comprehensive perioperative management similar to the protocols used in bariatric and metabolic surgery (BMS) are essential to provide a safe and minimally invasive option for severely obese patients [3].

Obesity is a recognized risk factor for the development of comorbid conditions such as cardiovascular disease, type 2 diabetes mellitus, malignancy, asthma, osteoarthritis, chronic back pain, obstructive sleep apnea, non-alcoholic fatty liver disease, and gallbladder diseases [4]. Current guidelines from the National Comprehensive Cancer Network® (NCCN®), the European Society of Medical Oncology (ESMO), and the Japanese Society for Cancer of the Colon and Rectum (JSCCR) have yet to be modified to adequately address the specific needs of obese patients with colorectal cancer. This gap highlights the importance of integrating multidisciplinary approaches to optimize outcomes for this population.

A randomized control study has also been reported that demonstrates the importance of prehabilitation in reducing postoperative complications and improving functional capacity after colorectal cancer surgery [5]. In this report, we describe a case in which safe, minimally invasive treatment was achieved through the implementation of multidisciplinary perioperative management and surgical techniques in collaboration with the BMS team from the preoperative phase. This case exemplifies the potential for improved patient outcomes when addressing the complexities associated with obesity in colorectal cancer treatment and underscores the need for evolving surgical guidelines to reflect the challenges and requirements of managing obese patients. By expanding our understanding of the interplay between obesity and colorectal cancer treatment, we can better tailor interventions to enhance safety and efficacy, ultimately improving the prognosis for this vulnerable patient population.

## Case presentation

**Figure 1:**
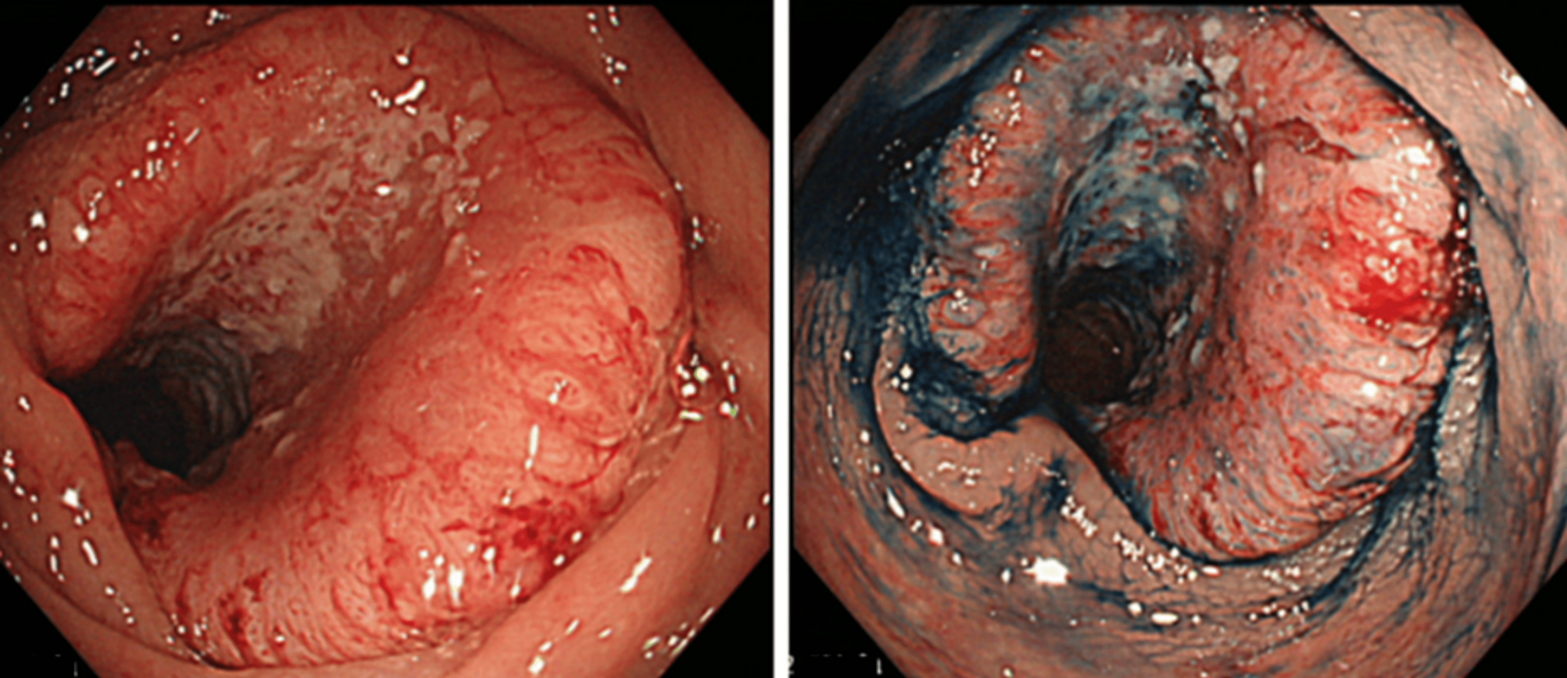
Preoperative colonoscopy A colonoscopy shows a type 2 tumor in the splenic flexure of the transverse colon.

The patient was a 45-year-old woman who became aware of bloody stools and visited the hospital. She underwent a colonoscopy, which found a type 2 tumor in the splenic flexure of the transverse colon, a biopsy resulted in a diagnosis of moderately differentiated adenocarcinoma (Figure 1).

**Figure 2:**
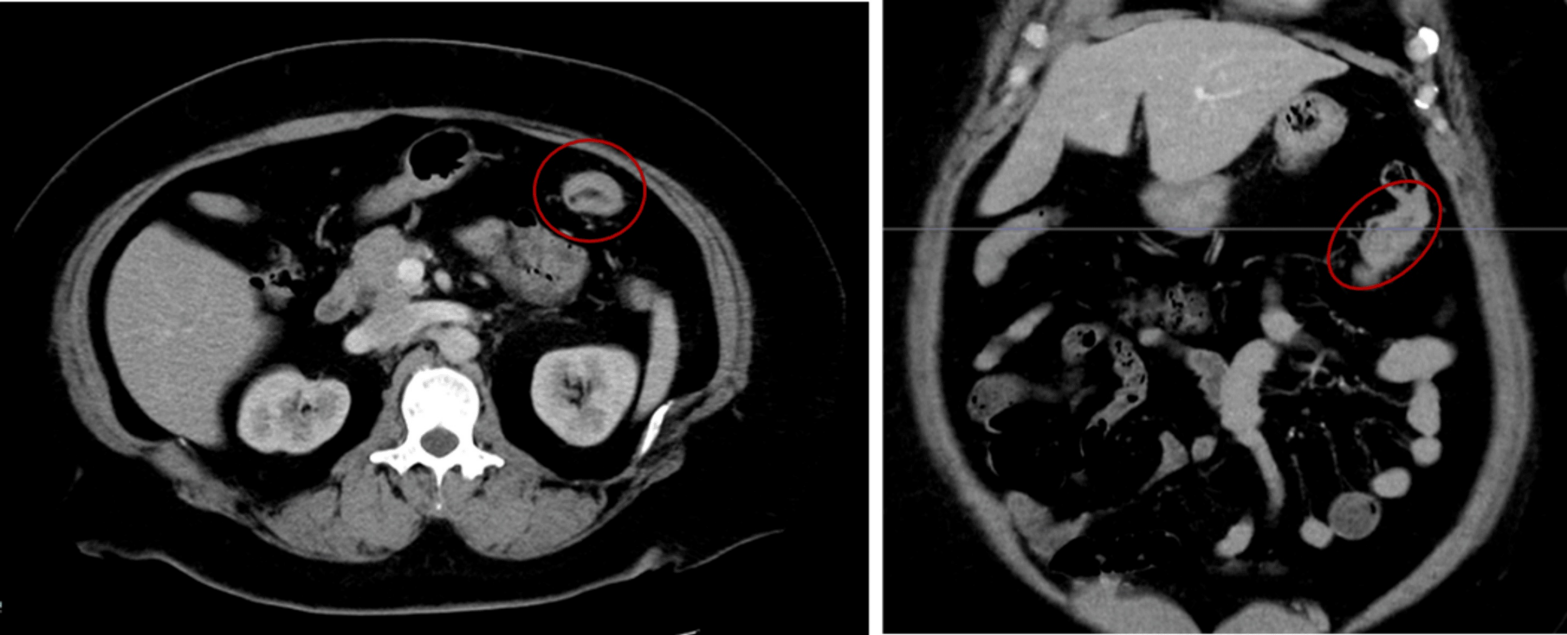
Preoperative CT CT shows transverse colon cancer in the splenic flexure (red-lined area) and no significant enlargement of the regional lymph nodes. CT: computed tomography

Contrast-enhanced computed tomography (CT) was performed, which showed no evidence of lymph node metastasis and distant metastasis, and a diagnosis of cT3N0M0 stage IIa transverse colon cancer was made (Figure 2).

The patient was 163 cm tall and weighed 134 kg, with a BMI of 50.43 kg/m^2^. She was severely obese and was referred to our hospital for surgical treatment. She had a history of bronchial asthma and laparoscopic cholecystectomy for cholelithiasis. Her pulmonary function tests showed that her ventilatory capacity was in the normal range, she was not anemic, and her liver and renal functions and glucose tolerance were in the normal ranges, but her serum carcinoembryonic antigen (CEA) level was abnormally high at 55.9 ng/ml.

Since the patient was severely obese with a BMI of 50.4 kg/m^2^ and was at high perioperative risk, the decision was made to provide active perioperative management from the preoperative period with the cooperation of the perioperative management center (PERIO), a perioperative management team that provides a safe and secure surgical and perioperative environment for patients undergoing surgery by performing interdisciplinary and inter-organizational work. PERIO noted difficulty in managing anesthesia due to poor intraoperative ventilation during laparoscopic surgery in the head-down position in a highly obese patient. The BMS team also advised a weight loss program before bariatric surgery for highly obese patients. Therefore, it was decided to offer preoperative weight loss treatment at a facility that offers a BMS preoperative weight loss program.

**Figure 3:**
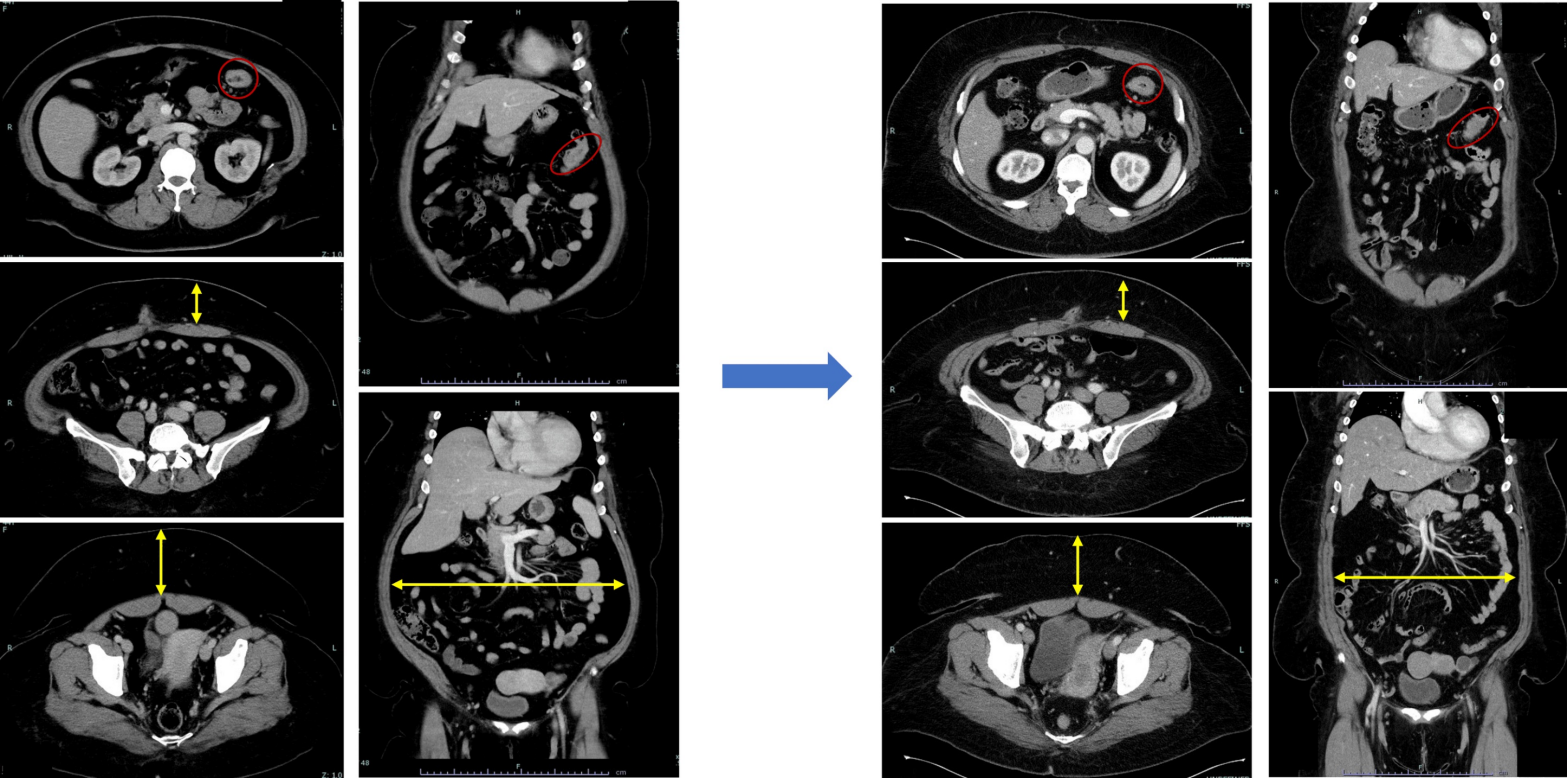
CT before and after weight loss treatment CT shows no obvious change in tumor and lymph node size before and after weight loss treatment (red-lined area), but CT shows a decrease in abdominal circumference and visceral fat (yellow arrows). CT: computed tomography

Normally, preoperative weight loss for BMS is performed for at least six months along with medical therapy, but in the present case, the patient had Stage IIa colon cancer and progression of the cancer had to be taken into consideration. Therefore, the duration of inpatient weight loss treatment was set to one month, with a target weight of 120 kg (-14 kg, -10% compared to pre-intervention weight), and inpatient weight loss treatment was performed. Preoperative weight loss treatment consisted of diet therapy using a formula diet and exercise rehabilitation focusing on aerobic exercise. Specifically, the caloric intake was set at 1000 kcal/day, with a formula diet designed to provide adequate nutrients while limiting carbohydrates and fats and ensuring sufficient proteins, vitamins, and minerals. The rehabilitation program focused on resistance exercise in the supine position and aerobic exercise on a bicycle ergometer since the patient had right knee pain and back pain due to severe obesity. In fact, calorie control of 1000 kcal/day and moderate exercise of rating of perceived exertion (RPE) 13-14 could be performed for one hour, and voluntary exercise of 9000-11000 steps/day, mainly Nordic walking, could be performed, resulting in weight loss of 200-600 g/day. As a result, the patient’s weight was successfully decreased from 134 kg to 121.5 kg (-12.5 kg, -9.3%), abdominal circumference was reduced from 140.2 cm to 128.5 cm (-11.7 cm), and BMI was reduced to 45.7 kg/m^2^. CT showed decreases in visceral fat, subcutaneous fat, and abdominal circumference as a result of weight loss treatment, and there was no evidence of tumor enlargement or enlarged regional lymph nodes (Figure 3).

With the patient’s BMI reduced to 45.7 kg/m^2^ by preoperative weight loss treatment, there was no intraoperative ventilatory disturbance. The patient underwent laparoscopic left hemicolectomy, D3 lymph node dissection, and intracorporeal anastomosis for transverse colon cancer at the splenic flexure, as scheduled. The operative time was 261 min, and the blood loss was 10 mL.

**Figure 4:**
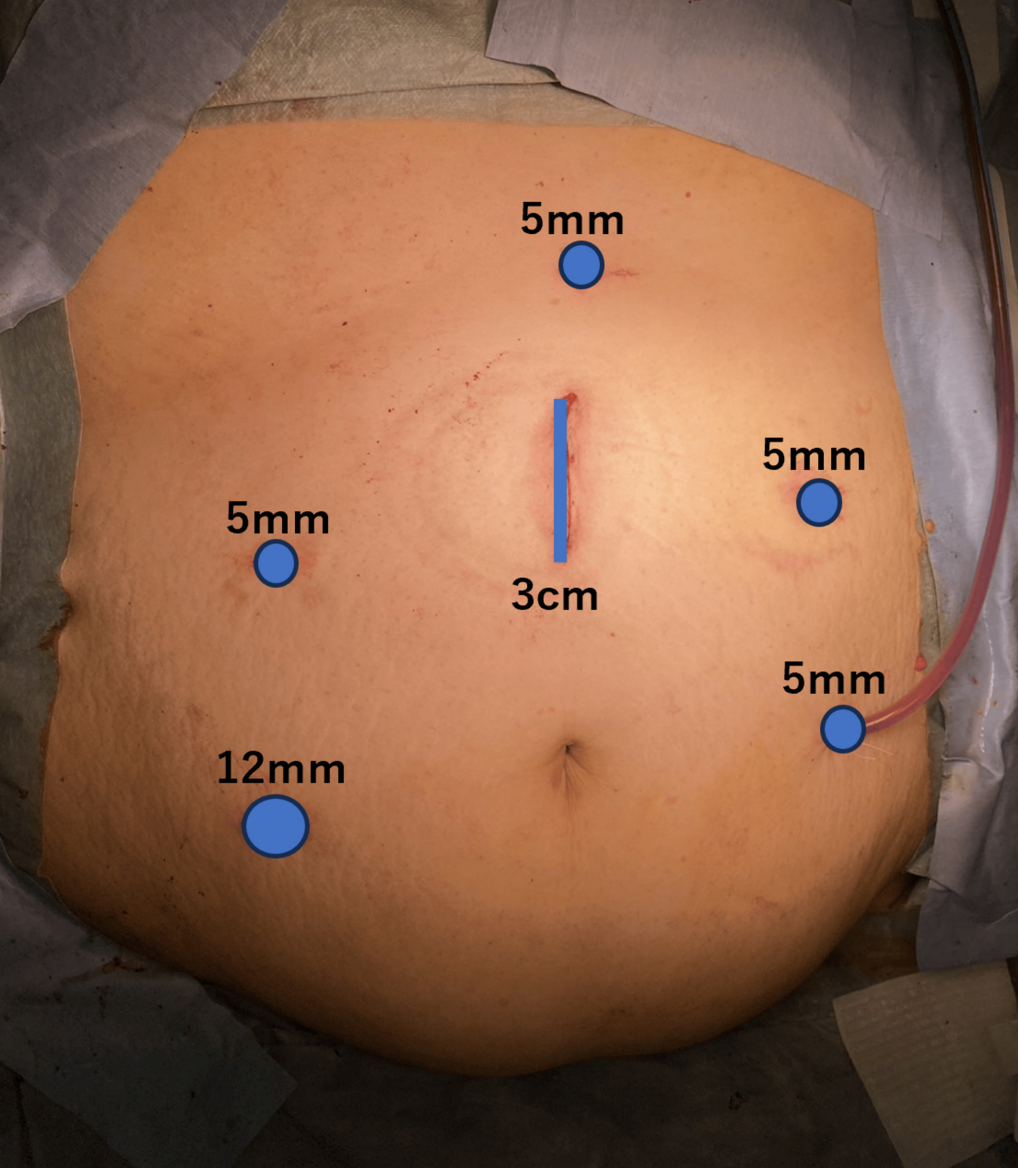
Laparoscopic port placement A small 3 cm incision is placed in the midline above the umbilicus, and the ports are positioned more cephalad than in the usual position.

The laparoscopic surgical procedures were completed safely in a highly obese patient by taking the following measures. First, for trocar selection, a long trocar was used in the lower abdomen, because subcutaneous fat restricts the range of motion of the trocar, and the Weck EFx Shield Fascial Closure System (Teleflex, Wayne, PA, USA.) was used for port closure. Next, a small incision for specimen removal was made above the umbilicus to insert the first trocar safely, and five additional trocars were also inserted above the umbilicus; their placement was determined based on the target vessels of the dissected lymph nodes in reference to preoperative CT. All trocars were placed more cephalad than usual, centered on the camera trocar above the umbilicus (Figure 4).

**Figure 5:**
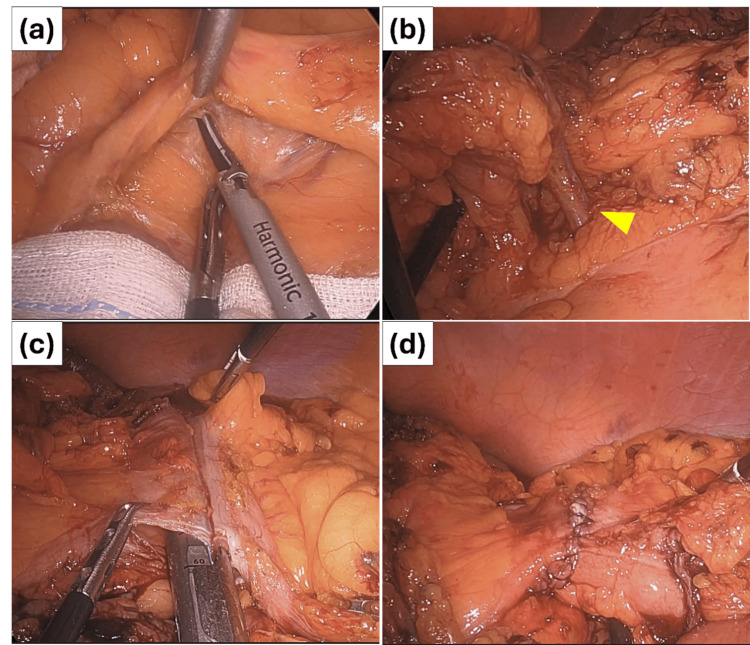
Intraoperative images (a) A retroperitoneal dissection was first performed using a laparoscopic gauze in the head-down position to ensure an adequate surgical view. (b) After lymph node dissection in the region of the middle colic artery (yellow arrowhead is the middle colic artery). (c) Overlap anastomosis using a laparoscopic linear stapler. (d) The hole where the stapler was inserted was closed with sutures, and the overlap anastomosis was completed.

With regard to surgical technique, efforts were made to recognize normal anatomy with careful dissection of adhesions, since obese patients often have intra-abdominal adhesions. It was difficult to secure a perfect surgical view due to visceral fat, and the minimum necessary surgical view was secured by positioning and laparoscopic gauze. Efforts were made to proceed expeditiously with retroperitoneal dissection in anticipation of a poor surgical view due to lipolysis or hemorrhage. An intracorporeal anastomosis was performed using the overlap technique with an endoscopic linear stapler, due to anticipated difficulty in elevating the intestine for an extracorporeal anastomosis (Figure 5).

**Figure 6:**
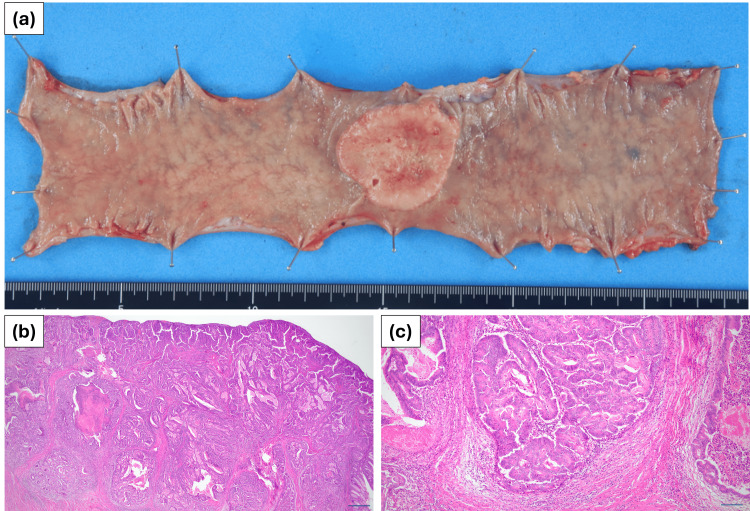
Histopathological findings Histopathological findings show a 47 mm-sized 6/7 circumscribed tumor in the transverse colon, with no lymph node metastasis, and a diagnosis of pT3 N0 M0 pStage IIA. (a) Macroscopic findings of transverse colon cancer. (b) Microscopic findings of a 20x HE-stained specimen (Line length indicates 1 mm). (c) Microscopic findings of a 100x HE-stained specimen (Line length indicates 0.2 mm)

The patient’s postoperative course was good. Postoperative rehabilitation and drinking of water were started the day after surgery, oral intake was started on the 4th postoperative day, and the patient was discharged home on the eighth postoperative day without any postoperative complications. The postoperative pathological diagnosis was colon cancer, T, Type 2, 47x42 mm^2^, 87.5% of circumference, tub2>tub1, pT3, INFb, Ly1a, V1a, BD1, EX(-), Pn0, pPM0, pDM0, pN0 (0/17) pStage IIA (UICC TNM classification) (Figure 6).

One year after surgery, there was no recurrence of colon cancer based on blood test results and contrast-enhanced CT findings.

## Discussion

The prevalence of obesity is increasing rapidly around the world, and according to the World Obesity Federation (WOF), the global obesity epidemic has nearly tripled since 1975. As of 2016, there were 1.307 billion overweight adults worldwide with a BMI between 25 kg/m^2^ and 30 kg/m^2^, and 671 million obese adults with a BMI of 30 kg/m^2^ or higher. One in five adults worldwide could be obese by 2025. One-third of obese adults have a BMI of 35 kg/m^2 ^or higher and are severely obese, requiring medical intervention [6]. In Japan, according to the National Health and Nutrition Survey Report by the Ministry of Health, Labor and Welfare, the proportion of obese people aged 20 years and over is 33.0% for men and 22.3% for women. By age group, the highest proportion of obese men is in their 40s, at 39.7%, followed by those in their 50s, at 39.2%, whereas the proportion of obese women increases in older age groups, with the highest proportion in their 60s, at 28.1%. Colorectal cancer surgery in severely obese patients is expected to be increasingly performed in the future. The need for perioperative management, including preoperative weight loss therapy, will also increase for colorectal cancer patients with severe obesity [7,8].

In a subgroup analysis of the JCOG 0404 trial, obesity with a BMI of 25 kg/m^2^ or greater was found to be associated with a poor prognosis and there are reports of a relationship between obesity and postoperative complications [1,2,9,10]. However, some reports of minimally invasive surgery for obese patients indicated that it can be performed as safely as in non-obese patients [11-13]. In general, the health risks associated with severe obesity include glucose intolerance, type 2 diabetes mellitus, dyslipidemia, hypertension, hyperuricemia/gout, coronary artery disease (myocardial infarction and angina pectoris), cerebral infarction (cerebral thrombosis and transient ischemic attack (TIA)), nonalcoholic fatty liver disease (NAFLD), abnormal menstruation/infertility, obstructive sleep apnea syndrome (OSAS), obesity hypoventilation syndrome, musculoskeletal disease (osteoarthritis), and obesity-related kidney disease. Of particular concern are heart failure, respiratory failure, venous thrombosis, OSAS, and obesity hypoventilation syndrome, which are actually important health problems in perioperative management [14]. Preoperative weight loss treatment is very important to reduce the risk of these perioperative complications. Therefore, in the present case, preoperative weight loss treatment was conducted based on the preoperative weight loss program used in BMS [3,15]. Although it is necessary to set a relatively short period of time for weight loss because of the risk of cancer progression, there is no recommended period of time. In the present case, the American College of Surgeons’ Case Triage Phase I on surgical postponement during a COVID-19 infection outbreak was used for the duration of weight loss [16]. Considering the high CEA of 55.9 ng/mL and the risk of lymph node metastasis, surgery was postponed to approximately three months after diagnosis. Therefore, the duration of inpatient weight loss treatment was approximately one month. For early-stage cancer, a weight loss period of about three months may be acceptable, and for advanced cancer with lymph node metastasis, a policy of preoperative chemotherapy and weight loss treatment may be considered as one treatment strategy to ensure safe surgery.

The weight loss program set the caloric intake at 1000 kcal/day as the diet. Since adequate protein intake would be difficult to obtain, a formula diet developed based on the very low-calorie diet (VLCD) theory was used to provide adequate nutrients even at low-calorie levels [15]. Exercise therapy consisted of a program of resistance exercise in the supine position and aerobic exercise on a bicycle ergometer (60-75 watts 15-25 minutes, RPE: 13-14) to prevent aggravation of musculoskeletal conditions due to right knee pain and low back pain caused by severe obesity. Thus, in patients with a limited period of weight loss, appropriate weight loss can be achieved by weight loss treatment under the supervision of an institution that can provide a weight loss program tailored to the patient’s condition. In fact, the patient successfully lost 12.5 kg (-9.3%), and her BMI decreased to 45.7 kg/m^2^ (-4.7 kg/m^2^). Furthermore, subcutaneous or oral formulations of GLP-1 receptor agonists are now approved by insurance as weight-loss treatments. In addition to conventional diet and exercise therapy, the use of weight-loss drugs such as GLP-1 receptor agonists may enable more effective preoperative weight loss [17,18].

Surgery for obese patients requires ingenuity in terms of instruments and techniques. First, especially in the lower abdomen, the use of a long trocar is necessary because the trocar range of motion is limited by the thickness of the subcutaneous fat, and it is difficult to close the port wound, so the use of a specialized abdominal closure device, the Weck EFx Shield Fascial Closure System (Teleflex), should be considered. Next, the umbilicus is often displaced caudally in obese patients due to subcutaneous fat. Therefore, the laparoscopic scope was positioned cephalad of the umbilicus where it is normally placed. Accordingly, all trocars were set cephalad of the usual position. The position of the first trocar was determined based on the target vessels of the lymph nodes to be dissected on preoperative CT. In this way, it is necessary to adjust the trocar position for each case. Regarding surgical techniques, the surgical view is limited by surrounding visceral fat such as mesenteric fat, making it difficult to secure a perfect surgical view. In some cases, the diaphragmatic compression of visceral fat causes poor ventilation, making it difficult to adequately maintain the head-down position for a long time. Therefore, it is necessary to communicate with the anesthesiologist during surgery to adjust the angle of the head-down position and use laparoscopic gauze to secure an adequate surgical view. In the present case as well, dissecting the retroperitoneum as widely as possible was tried first, considering the deterioration of the surgical view due to lipolysis and hemorrhage, as well as poor ventilation due to the head-down position. In terms of anastomosis, intracorporeal anastomosis should be considered, assuming difficulty in elevating the intestine and performing extracorporeal anastomosis. As previously reported, intracorporeal anastomosis is useful in obese patients [19,20].

## Conclusions

In the present case, the preoperative multidisciplinary weight loss treatment by the PERIO and BMS team was effective, and minimally invasive laparoscopic surgery could be safely completed without any ventilatory disturbance due to the head-down position. For patients with severe obesity, we will continue to provide safe, minimally invasive treatment by preoperative weight loss.
